# Genomic *EWS*-*FLI1* Fusion Sequences in Ewing Sarcoma Resemble Breakpoint Characteristics of Immature Lymphoid Malignancies

**DOI:** 10.1371/journal.pone.0056408

**Published:** 2013-02-18

**Authors:** Manfred Berger, Uta Dirksen, Andreas Braeuninger, Gabriele Koehler, Heribert Juergens, Manuela Krumbholz, Markus Metzler

**Affiliations:** 1 University Hospital Erlangen, Department of Pediatrics, Erlangen, Germany; 2 University Hospital Muenster, Department of Pediatric Hematology and Oncology, Muenster, Germany; 3 University Hospital Muenster, Department of Pathology, Muenster, Germany; Johns Hopkins University, United States of America

## Abstract

Chromosomal translocations between the *EWS* gene and members of the *ETS* gene family are characteristic molecular features of the Ewing sarcoma. The most common translocation t(11;22)(q24;q12) fuses the *EWS* gene to *FLI1*, and is present in 85–90% of Ewing sarcomas. In the present study, a specifically designed multiplex long-range PCR assay was applied to amplify genomic *EWS-FLI1* fusion sites from as little as 100 ng template DNA. Characterization of the *EWS-FLI1* fusion sites of 42 pediatric and young adult Ewing sarcoma patients and seven cell lines revealed a clustering in the 5′ region of the *EWS*-breakpoint cluster region (BCR), in contrast to random distribution of breakpoints in the *FLI1*-BCR. No association of breakpoints with various recombination-inducing sequence motifs was identified. The occurrence of small deletions and duplications at the genomic junction is characteristic of involvement of the non-homologous end-joining (NHEJ) repair system, similar to findings at chromosomal breakpoints in pediatric leukemia and lymphoma.

## Introduction

Reciprocal balanced chromosomal translocations are recurrent and specific somatic aberrations in a wide variety of tumors, and are particularly associated with hematological malignancies [1]. Sequence analysis of individual DNA breakpoints reveals fingerprints of different DNA recombination and repair mechanisms involved in chromosomal translocation, depending on the differentiation stage of the target cell at initiation. For example, chromosomal fusion sites of lymphoid malignancies are often linked to aberrant recombination activating gene (RAG)-mediated V(D)J recombination [2]. In multiple myeloma, incorrect class switch recombination by activation-induced deaminase (AID) during secondary antibody diversification induces translocations within the immunoglobulin switch regions [3]. Chromosomal breakpoints in therapy-related acute lymphoblastic leukemia are clustered in proximity to topoisomerase II binding sites of the *MLL* gene [4], [5]. By contrast, more immature leukemia cells show predominantly sequence-independent breakages [6].

Far fewer breakpoint DNA sequences have been deciphered from solid tumors to allow comparative analyses, although there is increasing evidence of recurrent chromosomal translocations in non-hematological tumors of both mesenchymal and epithelial origin [7]–[10]. One of the first solid tumors found to carry characteristic reciprocal chromosomal translocations was Ewing sarcoma [11]. Ewing sarcoma family (ES) are the second most common solid bone and soft tissue malignancies in children, adolescents and young adults. ES are associated with chromosomal rearrangements that result in the fusion of the *EWS* gene with one gene of the *ETS* family of transcription factors. The most common translocation found in ES is t(11;22)(q24;q12), which fuses the *EWS* gene with the *FLI1* gene. More than 85% of Ewing sarcoma patients carry a *EWS-FLI1* fusion sequence. In 10% of cases, the *EWS* gene is fused to the *ERG* gene resulting in the t(21;22)(q22;q12) translocation [12]. Fusion transcript type does not appear to imply a prognostic impact with current treatment regimens [13]. The frequency at which characteristic fusion genes were being identified led to the incorporation of molecular analysis, i.e., fluorescence *in situ* hybridization (FISH) or reverse transcription-PCR (RT-PCR), in the routine diagnostic workup for ES [14], [15]. The large introns within the genomic breakpoint cluster regions (BCRs), however, mean that identification of genomic breakpoints is limited by the amplification range of conventional PCR, and detection of genomic fusion sites is not yet a routine diagnostic test. The BCR of the *EWS* gene spans a 5.7 kb region between exon 7 and exon 11, and the BCR of the *FLI1* gene extends from exon 4 to exon 9, encompassing 38.2 kb.

Using primers covering both of the BCRs a nested multiplex long-range PCR (MLR-PCR) assay was established for reliable identification of *EWS-FLI1* fusion sites. Unlike leukemia, where diagnostic material is easily accessible from blood or bone marrow, diagnostic samples of ES- are usually obtained by tumor biopsy and, therefore, the amount of diagnostic DNA for genomic studies is often very limited. Using the MLR-PCR assay described herein, it was possible to identify genomic *EWS-FLI1* fusion sequences from as little as 100 ng DNA from the tumors of all the pediatric and young adult Ewing-sarcoma patients investigated.

In addition to diagnostic aspects, sequencing of genomic fusion sites is a prerequisite for detailed breakpoint characterization to identify breakpoint initiation mechanisms, and could also provide complementary information about the cellular origin of ES-.

## Materials and Methods

### Patients and Cell Lines

Genomic DNA was analyzed from t(11;22)(q24;q12) translocation-positive Ewing- sarcoma cell lines (n = 7), and 42 individuals (median age 14 years) whose cryopreserved tumor biopsies were sent to the Euro E:W:I:N:G:- 99 trial reference laboratory for pathological review and molecular diagnostics. Clinical and molecular parameters are summarized in Table 1. Informed consent was obtained from all patients or their legal guardians, in accordance with the declaration of Helsinki.

**Table 1:** Patientś characteristics and breakpoint coordinates of tumor samples and cell lines.

UPN	Age (y)	Sex	Localisation	RT - PCR	MLR - PCR	der22		der11		gains and losses	
Cell line				*EWS - FLI1*	*EWS - FLI1*	Break *EWS*	Break *FLI1*	Break *FLI1*	Break *EWS*	*EWS*	*FLI1*
											
1	4	F	Chest wall	ex7 - ex6	in8 - in5	21111	102511				
2	3	F	Chest wall	ex7 - ex6	in8 - in5	22095	92502	90259	22454	−358	−2242
3	7	M	Fibula	ex7 - ex6	in8 - in5	20804	101265	101204	20816	−11	−60
4	12	M	Pelvis	ex7 - ex6	in7 - in5	19984	93467				
5	21	M	Humerus	ex7 - ex6	in8 - in5	21707	92407				
6	15	F	Pelvis	ex7 - ex6	in8 - in5	21206	98678				
7	12	F	Lower leg	ex7 - ex6	in7 - in5	20171	106672				
8	15	M	Humerus	ex7 - ex6	in7 - in5	19231	92652	92789	18978	254	138
9	6	M	Fibula	ex7 - ex6	in7 - in5	20543	89407	89390	20547	−3	−16
10	15	M	Fibula	ex7 - ex6	in7 - in5	19957	96301	96268	19979	−21	−32
11	10	M	Femur	ex7 - ex6	in7 - in5	19581	95901	95903	19564	18	3
12	13	M	Pelvis	ex7 - ex6	in8 - in5	20988	94580				
13	10	M	Pelvis	ex7 - ex6	ex8 - in5	20663	98280	98274	20667	−3	−5
14	15	F	Talus	ex7 - ex6	in7 - in5	19480	101933	101204	20816	−1335	−728
15	16	M	Pelvis	ex7 - ex6	in7 - in5	19533	110315	110334	19538	−4	20
16	16	F	Lower leg	ex7 - ex6	in8 - in5	20987	106613				
17	18	M	Femur	ex7 - ex6	in7 - in5	20125	106653	106699	20055	71	47
18	20	F	Pelvis	ex7 - ex6	ex8 - in5	20604	99064	99071	20611	−6	8
19	8	M	Chest wall	ex7 - ex6	in8 - in5	20821	96410	96409	20848	−26	0
20	21	M	Os sacrum	ex7 - ex6	in7 - in5	19651	103185				
21	22	M	/	ex7 - ex6	in7 - in5	19144	110769	110770	19513	−368	2
22	19	F	Fibula	ex7 - ex5	in8 - in4	21330	79881				
23	30	M	Humerus	ex7 - ex6	in7 - in5	20127	98643	98651	20142	−14	9
24	18	M	Tibia	ex7 - ex6	in7 - in5	19939	107245	107250	19938	2	6
25	/	M	/	ex7 - ex6	in8 - in5	20860	93595				
26	12	M	Humerus	ex7 - ex5	in7 - in4	19846	84791	84793	19851	−4	3
27	13	M	/	ex10 - ex5	in10 - in4	24348	87199				
28	14	M	Ilium	ex7 - ex6	in7 - in5	19743	92762				
29	9	F	Pelvis	ex10 - ex8	in10 - in7	24275	113559	113559	24279	−3	1
30	25	M	Skapula	ex7 - ex5	ex11 - in4	24534	81974				
31	6	F	Tibia	ex7 - ex5	in7 - in4	19767	79357	79378	19750	18	22
32	11	M	Femur	ex7 - ex6	in7 - in5	20506	96596	96872	20329	178	277
33	4	M	Spine	ex7 - ex8	in7 - in7	20122	114396	114622	20119	4	227
34	15	F	Femur	ex10 - ex5	in10 - in4	24229	87490	87543	24057	173	54
35	16	M	Spine	ex7 - ex5	in8 - in4	21261	80293	79476	21555	−293	−816
36	5	M	Tibia	ex7 - ex8	in8 - in5	21260	93863				
37	16	M	Femur	ex10 - ex6	in10 - in5	24347	107895	107865	24342	6	−29
38	17	M	Chest wall	/	in9 - in7	23584	113386				
39	9	F	Fibula	ex7 - ex6	in8 - in5	20802	103255	103229	20802	1	−25
40	9	M	Humerus	/	in7 - in4	20326	85673	86256	19661	666	584
41	10	M	Skapula	/	in7 - in7	20029	115078	115070	20032	−2	−7
42	15	M	Femur	/	in7 - in5	19996	111261	111251	19859	138	−9
VH - 64				ex7 - ex5	In8 - In4	20874	82256	82445	20984	−109	190
TC - 71				ex7 - ex6	In7 - In5	20429	96791	96795	20433	−3	5
RD - ES				ex7 - ex5	In7 - In4	19680	85752	82518	19876	−195	−3233
WE - 68				ex7 - ex6	In8 - In5	21418	92974				
SKNMC				ex7 - ex6	In8 - In5	21511	100623				
A673				ex7 - ex6	In7 - In5	20283	103693	103692	20284	0	0
TC – 32				ex7 - ex6	In8 - In5	21354	88394	88388	21259	96	−5

Cell lines were cultured in RPMI medium supplemented with 10% fetal bovine serum, L-glutamine, and antibiotics at 37°C in 5% CO_2_ on collagen-coated flasks. DNA from cell lines and tumor tissues was isolated using a QIAamp® DNA Blood and Tissue Mini Kit (Qiagen GmbH, Hilden, Germany).

### Detection and Analysis of Genomic *EWS-FLI1* Fusion Sites

**Figure 1:**
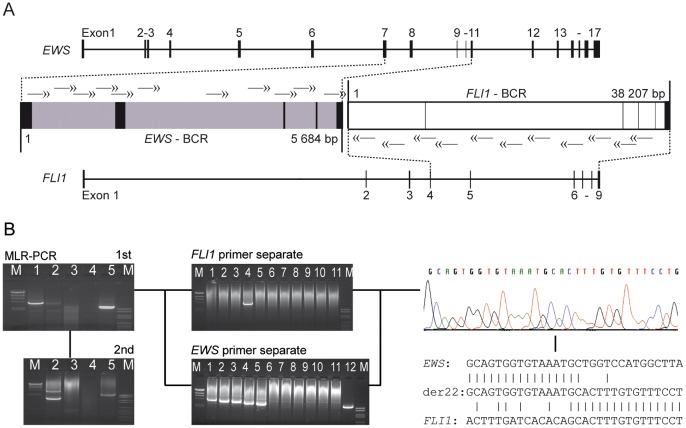
Genomic fusion site sequencing. (A) Genomic organization of the *EWS* and *FLI1* genes and corresponding breakpoint cluster regions (BCR). Nested primer sets for der22 are shown as double headed arrows. (B) Representative breakpoint sequencing workflow. Left: Gel electrophoresis of MLR-PCR products from two tumor samples in lane 1 and 2 (lane 3 negative control DNA; lane 4 ddH_2_O; lane 5 positive control DNA; M = DNA ladder). Center: Gel electrophoresis of single long-range PCR products from 1^st^ round MLR-PCR product of sample 1 (lane 1–11; lane 12 positive control) to identify *FLI1* and *EWS* primers next to the fusion sites and to reduce amplification product size for direct sequencing. Right: Sequencing of the shortest amplification product and alignment to *EWS* and *FLI1* reference sequences.

Genomic *EWS-FLI1* fusion sequences (derivative (der)22) were amplified by nested MLR-PCRs using 11 nested primer pairs covering both of the *EWS* gene and *FLI1* gene BCRs (Figure 1A). Due to the different BCR length of the *EWS* and *FLI1* genes, the average distance between primers was 500 bp and 3000 bp, respectively. In addition, an analogous primer set in the opposite direction was designed for the amplification of *FLI1-EWS* fusion sites (der11) (Figure S1). Primer sequences and positions are shown in Table S1.

All PCRs were performed using the AccuPrime™ *Taq* DNA Polymerase System (Invitrogen, Karlsruhe, Germany) according to the manufacturer’s instructions. For the first round, MLR-PCR 100 ng template DNA was combined with the most 5′ *EWS* sense primer and 11 antisense *FLI1* primers. If no specific amplification product was visible by gel electrophoresis, a second round MLR-PCR was carried out with corresponding internal primers and 1 µl of first round MLR-PCR product as template DNA (Figure 1B left side). To identify the *FLI1* primer positioned next to the fusion site and, therefore, responsible for product generation, a series of single LR-PCRs was set up with the most 5′ internal *EWS* sense primer, one of each internal antisense *FLI1* primers, and again, with first round MLR-PCR product as template DNA. Subsequently, the appropriate *FLI1* primer was used in a series of single LR-PCRs in combination with additional internal sense *EWS* primers to further reduce the size of the specific amplification product for direct sequencing on a Beckman Coulter CEQ 8800 Genetic Analysis System (Figure 1B middle, right side). Patient-specific breakpoints were confirmed by an independent PCR using specific primer sets next to the patient’s fusion site and 10 ng original tumor DNA.

### Statistical Breakpoint Analysis

Patient-specific fusion sequences were aligned to the NCBI reference sequences of *EWS* (NC_000022.10) and *FLI1* (NC_000011.9) using VectorNTI® software for sequence editing and analysis. Breakpoint and primer positions are numbered according to their positions in the reference sequences, starting with number one for the first nucleotide.

Repeat elements in the *EWS*- and *FLI1*-BCR were identified with the RepeatMasker tool (http://www.repeatmasker.org/). A Chi-square test was used to test for significant colocalization of patient-specific fusion sites and repeat elements or recombination-related DNA sequence motifs.

Components of the free software environment R (www.r-project.org) were applied to Kernel density analysis. Bandwidth selection was performed according to Sheather and Jones [16]. Clusters were defined as regions in which the lower limit of the 95% confidence band, determined by a bootstrapping procedure, was higher than a density function resulting from simulations at randomly distributed pseudo-breakpoints [17]. Both bootstrapping and simulations used 1000 permutations.

## Results

### Detection and Sequencing of Genomic *EWS-FLI1* Fusion Sites

A nested MLR-PCR assay was developed for detection of genomic *EWS-FLI1* and *FLI1-EWS* fusion sites from diagnostic tumor biopsy specimens. PCR conditions were optimized using DNA from seven *EWS-FLI1*-positive Ewing- sarcoma cell lines (Table 1). Sensitivity of the MLR-PCR was determined by a series of 2-fold dilutions of *EWS-FLI1-*positive A673 Ewing- sarcoma cells in *EWS-FLI1*-negative HL-60 cells. With the initial multiplex PCR, the *EWS-FLI1* fusion gene was detectable up to a 1∶64 dilution, corresponding to a tumor cell proportion of 1.6%. A subsequent quantitative real-time PCR, applying individually designed fusion site-specific primer and probe sets, allowed breakpoint detection with a sensitivity of 10^−4^–10^−5^ (data not shown).

A major advantage of the assay is the requirement for a minimal amount of DNA (100 ng) as both of the *EWS* and *FLI1* BCRs are covered by one single initial multiplex PCR. The genomic *EWS-FLI1* fusion sites (der22) were successfully amplified from all seven cell lines and 42 tumor samples (Table 1). The reciprocal genomic *FLI1-EWS* fusion sites (der11) were detected in 27/42 Ewing sarcoma patients and five out of seven cell lines (overall 65%). The lower detection rate of the reciprocal *FLI1-EWS* fusion site is in line with results from other recurrent chromosomal translocations and is attributable to large deletions or complex rearrangements on der11 [18]–[20].

Alignments of individual fusion sites to the reference sequences are shown in Figure S2. The breakpoint sequences were deposited in the NCBI GenBank (accession numbers JX266448 to JX266528).

### Distribution of Genomic Breakpoints

**Figure 2:**
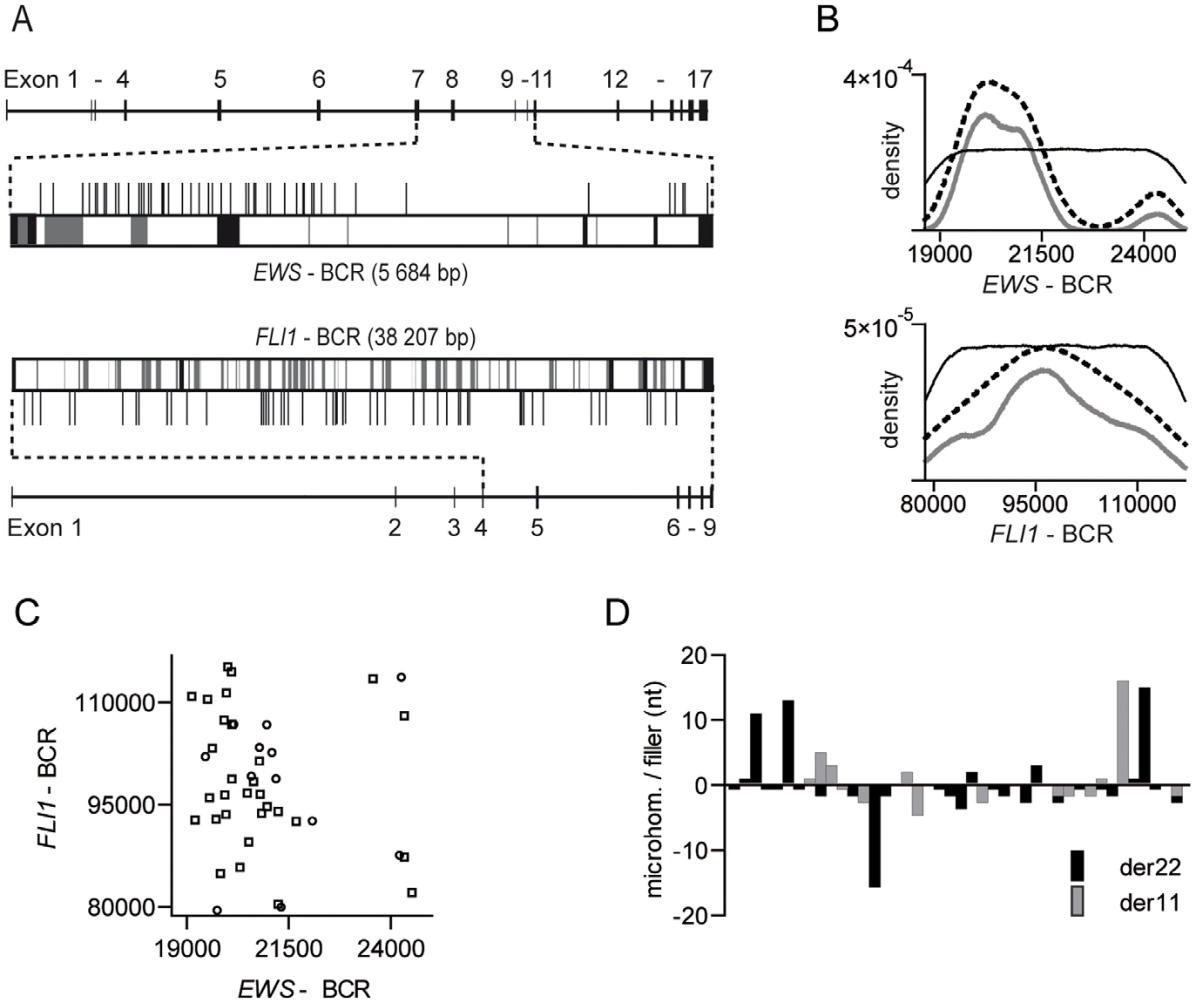
Breakpoint distribution in the BCR of *EWS* and *FLl1*. (A) Vertical bars above or below the breakpoint regions indicate individual breakpoint positions of Ewing sarcoma patients. Black boxes represent exons, and gray boxes correspond to repeat elements. (B) Results of Kernel density analysis (dashed line = breakpoint density; gray line = lower limit of 95% confidence band determined by bootstrapping procedure; black line = 95% confidence interval of a density function resulting from simulations at randomly distributed pseudo-breakpoints). X-axes indicate the BCR nucleotide positions within the respective reference gene. (C) Scatterblot of gender-specific *EWS-FLI1* breakpoints. Circles represent female, and squares represent male subjects. (D) Number of microhomologies and filler nucleotides at *EWS-FLI1* (der22; black bar) and *FLI1-EWS* (der11; gray bar) fusion sites. Each bar on the x-axis represents one individual.

Breakpoint distribution analysis of all 49 *EWS-FLI1* fusion sites revealed two cluster regions within the *EWS*-BCR. Forty-three of 49 breaks (88%) were located in a cluster region spanning from intron 7 to the 5′-region of intron 8; the remaining six breaks (12%) were positioned in a region spanning from intron 9 to exon 11, constituting a second cluster region (Figure 2A). Kernel density analysis confirmed this observation and showed a statistically significant overrepresentation of breakpoints within the cluster region at the 5′-region of the *ESW*-BCR (Figure 2B). Due to the low number of breakpoints in the second cluster at the 3′-region of the *EWS*-BCR no statistical significance was obtained by Kernel density analysis. By contrast, breakpoints in the *FLI1*-BCR were randomly distributed (Figures 2A and 2B). Localization of *EWS* breakpoints showed no significant correlation with the corresponding *FLI1* breakpoints (Figure 2C).

Patients with breakpoints in the smaller *EWS* cluster region revealed no differences with respect to age, gender (Figure S3A) and tumor localization. Similar results were obtained for the *FLI1*-BCR; no correlation between breakpoint localization and gender or age at time of diagnosis was observed (Figure S3B).

In 34/49 (69%) samples, genomic breakpoints within the *FLI1* gene resulted in an *EWS-FLI1* fusion transcript of *EWS* exons 1–7 fused to *FLI1* exons 6–9, representing Ewing sarcoma type 1. In seven out of 49 (14%), there was an *EWS-FLI1* fusion transcript of *EWS* exons 1–7 and *FLI1* exons 5–9, corresponding to Ewing sarcoma type 2. In 15 patients and four cell lines with genomic breakpoints located in *EWS* exon 8 or intron 8, exon 8 was spliced out at the transcriptional level (Table 1). The splicing out of exon 8 is essential to form a functionally active *EWS-FLI1* fusion transcript with an intact reading frame [21]. The frequencies of the different chimeric *EWS-FLI1* fusion transcript types in this study are consistent with published data [22].

### Sequence Analysis of *EWS-FLI1* and *FLI1-EWS* Fusion Sites

Genomic fusion sites of der22 and der11 were analyzed for colocalization with the following repeat elements and recombination-related DNA sequence motifs: *Alu* repeats, topoisomerase II binding sites, translin binding sites, chi-like sequences, heptamer/nonamer recombination signals, palindromic sequences, hypervariable minisatellite core sequence, hypervariable minisatellite recombination sequence, DNA polymerase frameshift hotspots, CpG islands, human replication origin consensus sequence, and repeat sequences such as low complexity, SINE/MIR, DNA/hAT-Charlie, and LINE/L2 (Table S2). No deviation between the expected and observed breakpoint within the DNA sequence motifs and repeat elements of *EWS*- and *FLI1*-BCR was observed (Table S2).

Detailed sequence analysis of the 49 identified *EWS-FLI1* and 32 *FLI1-EWS* genomic fusion sites revealed clean transitions between the two contributing genes in 30 cases (37%), 37 fusion sites (46%) featured small microhomologies (<17 bp), and another 14 breakpoints (17%) presented with filler nucleotides at the fusion sites (<16 bp) (Figure 2D). Patient UPN 5 showed an extended inverted insertion of a *FLI1* gene segment between the 5′ to 3′ *EWS* and *FLI1* portion at the genomic breakpoint.

These findings are comparable to breakpoint structures observed in hematological malignancies. Immature leukemia cells in particular show predominantly sequence-independent breakage with small microhomologies or deletions at the fusion sites [4], [6].

## Discussion

Chromosomal fusion of the *EWS* gene to one member of the *ETS* family of transcription factors is the genetic hallmark of ES-. Detection of the chromosomal translocation by FISH, or amplification of the resulting fusion transcripts by RT-PCR, has become a well-established diagnostic component for molecular confirmation of histopathological tumor classification. Chromosomal breakage and re-fusion, however, occur in large intronic parts of the respective genes. Although the large size of the BCRs complicates the amplification of the breakpoint-spanning site, identification of the individual intronic DNA fusion sequence may unravel additional information associated with mechanisms involved in the rearrangement formation.

DNA-based tumor diagnostics has general advantages and disadvantages. The chemical stability of DNA facilitates storage and transport of patient material, and in contrast to RT-PCR methods, enables the detection of fusion genes independent of their gene expression. As a complementary diagnostic tool, DNA-based minimal residual disease detection could improve the quantification of resting residual tumor cells from different specimens including bone marrow, peripheral blood stem cell collection products, and paraffin-embedded, fixed-tissue sections. Fusion of *EWS* to one of its partner genes is an early event in tumorigenesis and an essential oncogenic factor for maintaining the malignant transformation of ES- [23], [24]. In contrast to secondary genetic aberrations occurring during clonal evolution and contributing to the genetic heterogeneity within an individual ES- [25], the genomic fusion site remains a common and consistent molecular marker of tumor cells, unaffected by clonal selection from therapeutic intervention, and is currently evaluated as an additional tool for tumor cell quantification during treatment.

A major obstacle in quantifying DNA breakpoints in ES is the identification of the genomic *EWS-FLI1* fusion sequence, because of large intronic regions within the BCRs. A classical approach, using multiple single PCRs to cover the complete BCRs, requires large amounts of patient material. This aspect and its consequent limit on the availability of adequate material for additional molecular diagnostics are probably the main reasons why only very few studies on genomic *EWS-FLI1* fusion sequences are published, in contrast to numerous studies on *EWS*-*FLI1* transcripts and genomic breakpoints in fusion genes in diseases with more freely available material, e.g., acute leukemia [13], [26]. Two studies have identified the genomic fusion sequence in a total of eight individual cases of ES on the basis of transcript amplification, using primers specifically designed for the region of interest [27], [28]. A larger cohort of 77 Ewing- sarcoma patients was analyzed by Zucman-Rossi et al. using 15 single PCRs, but no patient characteristics are available and primer sequences have not been made permanently available [18].

In the present study, to overcome the problem of high DNA consumption, a nested MLR-PCR assay was established for reliable amplification of genomic *EWS-FLI1* fusion sequences. Highly stringent primer selection and the use of advanced polymerases facilitated the development of a detection assay based on a single initial MLR-PCR. Thus, only minimal patient material is required to extract template DNA (100 ng), enabling the detection of tumor genomic fusion sequences from small tumor samples, e.g., fine needle biopsies. Using this assay *EWS-FLI1* fusion sequences were identified from all 42 pediatric and young adult ES patients investigated. In addition, an analogous primer set was designed for detection of the second most common fusion gene in ES, the *EWS-ERG* gene. The assay can be readily adapted to include rare fusion partner genes occurring in the remaining 2–5% of cases [12].

The patient-specific breakpoints are distributed in two subclusters within the *EWS*-BCR, and this is in line with results from the cohort studied by Zucman-Rossi et al. [18]. Despite searching for an extensive spectrum of sequence motifs and repeat elements, no DNA motif associated with either of the breakpoint clusters or with a significant subgroup of Ewing sarcoma was identified. However, small microhomologies and filler nucleotides at the fusion sites, as well as deletions or insertion of several nucleotides in the corresponding chromosomal derivatives (der22 and der11), resemble the characteristics of non-homologous end-joining (NHEJ) repair, and suggest that NHEJ repair is involved in translocation formation in ES [29].

A comparison of genomic breakpoint features of ES- with the far more extensively characterized breakpoints in leukemia and lymphoma reveals a number of similarities. In cells from hematological malignancy, different DNA double-strand break and repair mechanisms are associated with the lineage and differentiation stage of the cell population. Sequence-independent breakages as observed in ES- are also characteristic for immature lymphoid malignant cells [6]. This subtype of leukemia cells shows small microhomologies or non-template insertions, as well as small deletions and duplications at the chromosomal breakpoint regions, indicating that NHEJ repair is involved in breakpoint initiation [4], [30]–[32].

This observation of reciprocal balanced translocations with an NHEJ repair signature indicates molecular genetic similarities to mesenchymal tumors. It further supports results from functional and molecular genetic studies characterizing ES- by global gene expression profiling, which propose that ES- has its cellular origin in early mesenchymal progenitors [33], [34].

In summary, in this study, genomic *EWS-FLI1* fusion sequences were identified in a cohort of pediatric and young adult Ewing sarcoma patients with a specifically designed MLR-PCR assay requiring only minimal patient DNA. Detailed characterization of the genomic fusion sites revealed similarities to fusion site characteristics identified in immature lymphoid leukemia cells, and suggests that NHEJ repair mechanisms are involved in breakpoint initiation.

## Supporting Information

Figure S1 **Genomic organization of the** ***FLI1*** **and** ***EWS*** **genes and corresponding breakpoint cluster regions (BCR).** Nested primer sets for der11 are shown as double headed arrows.
(TIF)

Figure S2 **Alignments of the patients’ specific fusion sequences to** ***EWS*** **and** ***FLI1*** **reference sequences.**
(DOC)

Figure S3 Scatterblots of the distribution of (A) *EWS* breakpoints and (B) *FLI1* breakpoints in reference to the age at time of diagnosis. Circles represent female, squares represent male subjects. Y-axes indicate the BCR nucleotide positions within the respective reference gene.
(TIF)

Table S1 Sequences of primers used in nested MLR-PCR assay.
(DOC)

Table S2 Summary of sequence motifs and repeat elements tested for association with breakpoint localization.
(DOC)
