# Supplementary figures and images for: Genomic EWS-FLI1 Fusion Sequences in Ewing Sarcoma Resemble Breakpoint Characteristics of Immature Lymphoid Malignancies
Source: PLoS One. 2013 Feb 18;8(2):e56408. doi: 10.1371/journal.pone.0056408 (PMC3575406; doi:10.1371/journal.pone.0056408)

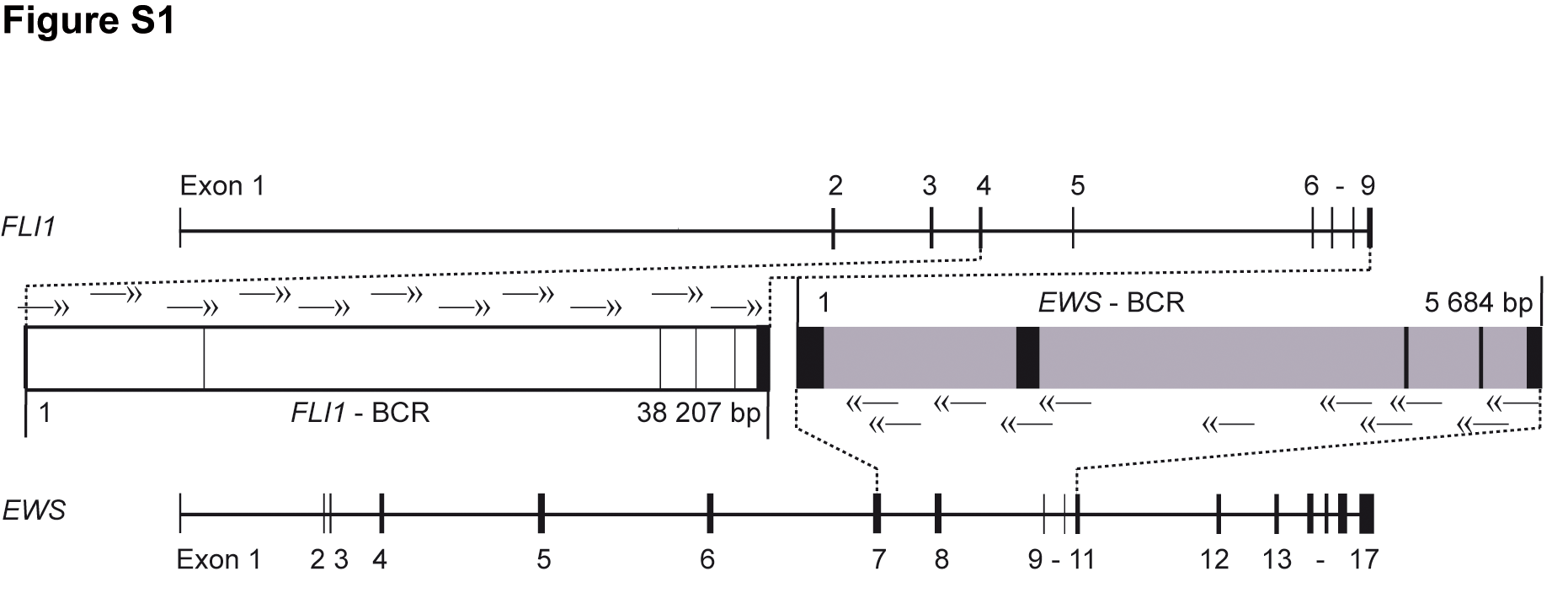

Supplement: Figure S1 — Genomic organization of the FLI1 and EWS genes and corresponding breakpoint cluster regions (BCR). Nested primer sets for der11 are shown as double headed arrows. (TIF) [file pone.0056408.s001.tif]

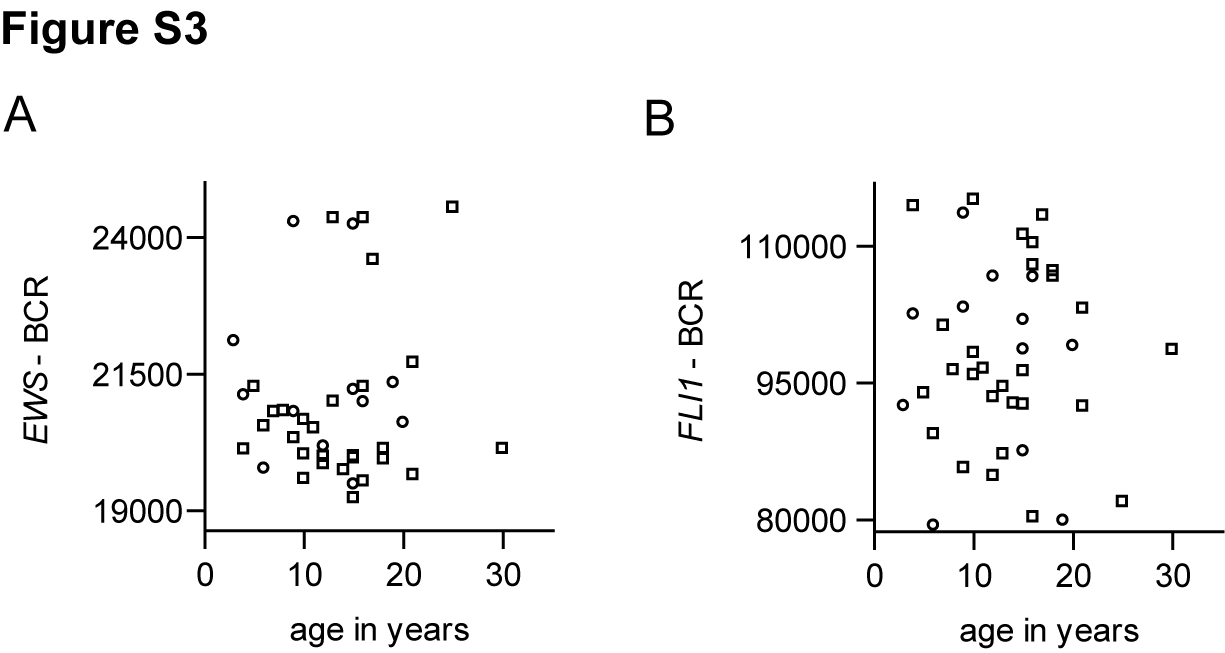

Supplement: Figure S3 — Scatterblots of the distribution of (A) EWS breakpoints and (B) FLI1 breakpoints in reference to the age at time of diagnosis. Circles represent female, squares represent male subjects. Y-axes indicate the BCR nucleotide positions within the respective reference gene. (TIF) [file pone.0056408.s003.tif]
