# Supplementary material for: Genomic EWS-FLI1 Fusion Sequences in Ewing Sarcoma Resemble Breakpoint Characteristics of Immature Lymphoid Malignancies
Source: PLoS One. 2013 Feb 18;8(2):e56408. doi: 10.1371/journal.pone.0056408 (PMC3575406; doi:10.1371/journal.pone.0056408)
Supplement: Figure S2 — Alignments of the patients’ specific fusion sequences to EWS and FLI1 reference sequences. (DOC) [file pone.0056408.s002.doc]

**Figure S2**

Genomic fusion sites

*EWS*: CTAATGACCCTAAGTTGTTAACTAACTGCCTTTGAATGGTTGTCTTGAAA

||||||||||||||||||||||||| ||| |||

Patient 1 der22: CTAATGACCCTAAGTTGTTAACTAATGGCCACATATGCTACCTCTCAGGC

| | | || ||||||||||||||||||||||||||

*FLI1*: TTCAATTTCTATTAAAATTCTTACATGGCCACATATGCTACCTCTCAGGC

*EWS*: AGACTTTGCTAGTAGAGGGGGCCCCTCTTTCTGGATGAGAAGTCTCTCTT

||||||||||||||||||||||||| |||

Patient 2 der22: AGACTTTGCTAGTAGAGGGGGCCCCCACAACTGTACGTTCGTACATCTCA

| | | | | ||||||||||||||||||||||||

*FLI1*: ATTGATGCATGATGTTACTGATTTAGACAACTGTACGTTCGTACATCTCA

*FLI1*: TATTGGGCTTCAGATATAGGGTTTAGTTTAGAAACTGGTTTAGCCAAGTT

||||||||||||||||||||||||| | | | | ||

Patient 2 der11: TATTGGGCTTCAGATATAGGGTTTAAGCATAATGAAAAGTGGGACTAGAC

|| |||| | |||||||||||||||||||||||||

*EWS*: TACCCGTACTCAGTACTCAGCCGGCAGCATAATGAAAAGTGGGACTAGAC

*EWS*: CCTAATTTTGTTTCATCCATAGGATTTTCAGTGGAAAGAAGGGACTGAAA

||||||||||||||||||| | | ||| | | | | |

Patient 3 der22: CCTAATTTTGTTTCATCCACAAGAATTTAAACTTTATCAGTCAAAATCCA

|| | || || | | | |||||||| |||||||||||

*FLI1*: GAAAAGATGGTAAAATTAAGTGGGACAATTACTTTATCTGTCAAAATCCA

*FLI1*: GGCTTAACATGCCTCTTTGATAAAGTTTAAATTCTTGTATTTGGCATATT

||||||||||||||||||||||||| | || | | | |

Patient 3 der11: GGCTTAACATGCCTCTTTGATAAAGGTGGAAAGAAGGGACTGAAAGACAT

| | | |||||||||||||||||||||||||

*EWS*: TTTTGTTTCATCCATAGGATTTTCAGTGGAAAGAAGGGACTGAAAGACAT

*EWS*: GGCAACATAGTGAGACCCTGTCTTTTTAAAGATAAATAAAGATTCTTTAA

||||||||||||||||||||||||| | | | ||

Patient 4 der22: GGCAACATAGTGAGACCCTGTCTTTCAAGTCCGGGCCTTTGGTGTGTTCT

| | ||||||||||||||||||||||||||

*FLI1*: CCAGGTCATGAAGCAGAACAAGACTCAAGTCCGGGCCTTTGGTGTGTTCT

*EWS*: TTTTTTAAAAAACAGAATGAACTTCAAAATTAAAGTTGATTTTTAACTTC

|||||||||||| |||||||||||| || || | | | |

Patient 5 der22: TTTTTTAAAAAATAGAATGAACTTCTTTATCAATATACCAGTAGGGCCTG

| | | ||||||||||||||||||||||||||

*FLI1*: TACAGTCTCTTCACTTACCTATACCTTTATCAATATACCAGTAGGGCCTG

*EWS*: TTGTCCCCAACTTCTGTTTGTACTCAGAATCGTTGAACATATGAAGATTT

||||||||||||||| |||| | | |

Patient 6 der22: TTGTCCCCAACTTCTATTTGACTGATAAGCGTGGACAAACGGGCTTCAGA

| || || || | | |||||||||||||||||

*FLI1*: GAGGGAGAGGAAGCTTCTTCCATGCCACATGGAGACAAACGGGCTTCAGA

*EWS*: CACGGGGATTTCTCAACAACTCAAAGGAGTAGTGTTGATTTATGGAGCTC

||||||||||||||||||||||||| | || || |

Patient 7 der22: CACGGGGATTTCTCAACAACTCAAATGTCCAGAAACAGCTTTCGCCTTCC

|| || || ||| | ||||||||||||||||||||||||||

*FLI1*: CATTGGTATGTCTATGAGAGGGTCATGTCCAGAAACAGCTTTCGCCTTCC

*EWS*: GTTTCACTCTTGGGGCTGAGGCTGGAGTACAGTGGTGCCATCTCAGCTCA

||||||||||||||||||||||||| || | ||

Patient 8 der22: GTTTCACTCTTGGGGCTGAGGCTGGCTGGAGCCAGGGCTACTGACTTTCC

| | | | | | |||||||||||||||||||||||||

*FLI1*: TGGCCTTTTCAAGTGTGTAAGAGTCCTGGAGCCAGGGCTACTGACTTTCC

*FLI1*: AAACCCTGCCGGAGGCCTTCCCAGCACACTCAGCTGGCCCAGCAGTGTGC

||||||||||||||||||||||||| | | | || || |

Patient 8 der11: AAACCCTGCCGGAGGCCTTCCCAGCTGAGTAGCTATGGTCAACAAAGCAG

| | || | |||| ||||||||||||||||||||||||

*EWS*: GGGCAACCGAGCAGCTATGGACAGCAGAGTAGCTATGGTCAACAAAGCAG

*EWS*: GTACATCAGGCAGTGGTGTAAATGCTGGTCCATGGCTTACAGATGTGACT

||||||||||||||||||||||||| | | |

Patient 9 der22: GTACATCAGGCAGTGGTGTAAATGCACTTTGTGTTTCCTGTTAACAATTT

| || | | |||||||||||||||||||||||||||

*FLI1*: AAGGTTACAACTTTGATCACACAGCACTTTGTGTTTCCTGTTAACAATTT

*FLI1*: GCTGGCTGTCTTGCCCAAGGTTACAACTTTGATCACACAGCACTTTGTGT

||||||||||||||||||||||| || || || | | | || |

Patient 9 der11: GCTGGCTGTCTTGCCCAAGGTTATAAATTCCATGGCTTACAGATGTGACT

| | ||||||||||||||||||||||

*EWS*: GTACATCAGGCAGTGGTGTAAATGCTGGTCCATGGCTTACAGATGTGACT

*EWS*: TGAGACCAGGAGTTTGAGACCACCCTGGGCAACATAGTGAGACCCTGTCT

||||||||||||||||||||||||| ||||| |

Patient 10 der22: TGAGACCAGGAGTTTGAGACCACCCGTCTGCTTTCTATCTGACCCTCTTC

| | | | |||||||||||||||||||||||||

*FLI1*: TTTGAAATTCCTGAGTTAATGTTCTGTCTGCTTTCTATCTGACCCTCTTC

*FLI1*: CTCTCTATTTTATTTTTTAAAAACTGTTTTCCTTTGAAATTCCTGAGTTA

||||||||||||||||||||||||| || ||| |||

Patient 10 der11: CTCTCTATTTTATTTTTTAAAAACTTCAGTCTTTTTAAAGATAAATAAAG

| | | | | | ||||||||||||||||||||||

*EWS*: ACCACCCTGGGCAACATAGTGAGACCCTGTCTTTTTAAAGATAAATAAAG

*EWS*: ATGGCATTCCAGCTAACATCTTGGATGCCCCAGTGAAGATTTCAGTGCTT

||||||||||||||||||||||||| | | || |

Patient 11 der22: ATGGCATTCCAGCTAACATCTTGGAAATACTGTGAGGTAATCTCCTGTTA

| | | || | |||||||||||||||||||||||||

*FLI1*: TGTGTACCCAAAGGAAAAGGCCTGTAATACTGTGAGGTAATCTCCTGTTA

*FLI1*: GTACCCAAAGGAAAAGGCCTGTAATACTGTGAGGTAATCTCCTGTTATTT

||||||||||||||||||||||||| | | | | ||

Patient 11 der11: GTACCCAAAGGAAAAGGCCTGTAATCCAGCTAACATCTTGGATGCCCCAG

| || || | | | | ||||||||||||||||||||||||||

*EWS*: GGACACATCTTTAGGGCATGGCATTCCAGCTAACATCTTGGATGCCCCAG

*EWS*: CTTCGTTGGGTCGGGGAGAACAAAGAATGGTTTTGAAACCAGAGCTTTCA

||||||||||||||||||||||||| | | | | | |

Patient 12 der22: CTTCGTTGGGTCGGGGAGAACAAAGGGAGACACACCTAGGAAATATGAAG

| | |||||||||||||||||||||||||||

*FLI1*: ACAAAGCAAAAAAACAACTATTTAGGGAGACACACCTAGGAAATATGAAG

*EWS*: TTCCGGACCAGGAGAGAACCGGAGCATGAGTGGCCCTGATAACCGGGGCA

|||||||||||||||||||||||| || | ||

Patient 13 der22: TTCCGGACCAGGAGAGAACCGGAGTTCCTGTAGTCCCCTCCCCACCTAGG

| | | |||||||||||||||||||||||||||

*FLI1*: GAAACAGAAACAGAAGAGAGCCTGTTCCTGTAGTCCCCTCCCCACCTAGG

*FLI1*: CTAGTATGAAACAGAAACAGAAGAGAGCCTGTTCCTGTAGTCCCCTCCCC

||||||||||||||||||||||||| | | | | |

Patient 13 der11: CTAGTATGAAACAGAAACAGAAGAGTGGCCCTGATAACCGGGGCAGGGGA

| | | ||||||||||||||||||||||||||||

*EWS*: GACCAGGAGAGAACCGGAGCATGAGTGGCCCTGATAACCGGGGCAGGGGA

*EWS*: CAGTCATGAGCCACTGCGCCCAGCCACGTTTGGAGTTTTTGAACAGGGGG

||||||||||||||||||||||||| | |||| | ||

Patient 14 der22: CAGTCATGAGCCACTGCGCCCAGCCCTAAAAAGCATTTTCATATGCATGG

||| | ||||||||||||||||||||| |||||||||||||||||||||

*FLI1*: CAGGCGTGAGCCACTGCGCCCAGCCCTAGAAAGCATTTTCATATGCATGG

*FLI1*: GGCTTAACATGCCTCTTTGATAAAGTTTAAATTCTTGTATTTGGCATATT

||||||||||||||||||||||||| | || | | |

Patient 14 der11: GGCTTAACATGCCTCTTTGATAAAGGTGGAAAGAAGGGACTGAAAGACAT

| || |||||||||||||||||||||||||

*EWS*: TTTTGTTTCATCCATAGGATTTTCAGTGGAAAGAAGGGACTGAAAGACAT

*EWS*: ACTCTTTGCCATTGTTTGCTTTTGGAATCCAGGACACATCTTTAGGGCAT

||||||||||||||||||||||||| | ||

Patient 15 der22: ACTCTTTGCCATTGTTTGCTTTTGGGGCTGAAGGGCATGGTTGTGTTTCA

|| | |||||||||||||||||||||||||||

*FLI1*: TAATTTGTTTGAGAGGCTCAGAAGGGGCTGAAGGGCATGGTTGTGTTTCA

*FLI1*: CAGAAGGGGCTGAAGGGCATGGTTGTGTTTCACCCCGTCAGACTCTGTTG

||||||||||||||||||||||||| ||| | || ||

Patient 15 der11: CAGAAGGGGCTGAAGGGCATGGTTGCAGGACACATCTTTAGGGCATGGCA

| | |||||||||||||||||||||||||

*EWS*: TTTGCCATTGTTTGCTTTTGGAATCCAGGACACATCTTTAGGGCATGGCA

*EWS*: TACTTCGTTGGGTCGGGGAGAACAAAGAATGGTTTTGAAACCAGAGCTTT

|||||||||||||||||||||||||| | | | || |

Patient 16 der22: TACTTCGTTGGGTCGGGGAGAACAAAACCAGGTTAAAAGCTTCACACAGA

| | | | | ||||||||||||||||||||||||

*FLI1*: CAAAGATGTTTGGCCATATTTCTAGGACCAGGTTAAAAGCTTCACACAGA

*EWS*: ATCTTCAGAATTTAGGATCATTGCTCTTTTTAAATTACATTCTATCCACG

||||||||||||||||||||||||| | | | || | | |

Patient 17 der22: ATCTTCAGAATTTAGGATCATTGCTGTATGTCTATGAGAGGGTCATGTCC

| || | | |||||||||||||||||||||||||

*FLI1*: TCACACAGAAGCTAAGTTAACATTGGTATGTCTATGAGAGGGTCATGTCC

*FLI1*: CCAGAAACAGCTTTCGCCTTCACAACAGAGACTTTGCGGAAAAAAATCTC

||||||||||||||||||||| | | | | |

Patient 17 der11: CCAGAAACAGCTTTCGCCTTCCAATTTGCAGTTCTTCTGTATGGAGAGAG

| | | || | |||||||||||||||||||||||||||

*EWS*: AAAAATAATCCTCCTCACAGAATATTTGCAGTTCTTCTGTATGGAGAGAG

*EWS*: TTCATTCCGACAGGACCACCCCAGTAGCATGGGTGTTTATGGGCAGGAGT

||||||||||||||||||||||||| | || | || ||

Patient 18 der22: TTCATTCCGACAGGACCACCCCAGTGGTGTGTTCCTATAGTCCCAGCTAC

| | | | | ||||||||||||||||||||||||||||

*FLI1*: ATAAACAAAAGTAGCCAGGTGTAGTGGTGTGTTCCTATAGTCCCAGCTAC

*FLI1*: CAAAAGTAGCCAGGTGTAGTGGTGTGTTCCTATAGTCCCAGCTACTCCAG

||||||||||||||||||||||||| | || |

Patient 18 der11: CAAAAGTAGCCAGGTGTAGTGGTGTTTATGGGCAGGAGTCTGGAGGATTT

| || ||| | ||||||||||||||||||||||||||||||

*EWS*: AGGACCACCCCAGTAGCATGGGTGTTTATGGGCAGGAGTCTGGAGGATTT

*EWS*: TAATTTTGTTTCATCCATAGGATTTTCAGTGGAAAGAAGGGACTGAAAGA

||||||||||||||||||||||||| |||| | | | |

Patient 19 der22: TAATTTTGTTTCATCCATAGGATTTCAGAGAGAAACAGAGATCGGGGGAG

| | | | | | |||||||||||||||||||||||||

*FLI1*: AGAGAGAGAGAAAGAACGAGGGTGACAGAGAGAAACAGAGATCGGGGGAG

*FLI1*: AGAGAGAGAGAAAGAACGAGGGTGACAGAGAGAAACAGAGATCGGGGGAG

||||||||||||||||||||||||| | || |

Patient 19 der11: AGAGAGAGAGAAAGAACGAGGGTGATAATTTCTGCATTTCCATGGACAAT

|| || |||||| | | |||||||||||||||||||||||||

*EWS*: GAAGGGACTGAAAGACATAAGAAATTAATTTCTGCATTTCCATGGACAAT

*EWS*: ATGCCTTGGCAAAAAAGAAAAGAGATACTAATTGATCAGAAAAAATGTTT

||||||||||||||||||||||||| | ||| | | |

Patient 20 der22: ATGCCTTGGCAAAAAAGAAAAGAGAGGCAAATCATGGGAATTGTTGGGAT

| | | || | | | ||||||||||||||||||||||||||

*FLI1*: AAAGCATTTCAGACATAGGTCAAAAGGCAAATCATGGGAATTGTTGGGAT

*EWS*: GAGAGAAAACCAAATAAGAATGAATGTGTTTAGAGTTTTTTTGTGGGGTT

||||||||||||||||||||| ||| | |

Patient 21 der22: GAGAGAAAACCAAATAAGAATCAATAGAAAAGCCCGAGAGCCCTCAGAGA

|| | || | |||||||||||||||||||||||||||||||

*FLI1*: CAGCCCAGCCCTCTCCACTATCAATAGAAAAGCCCGAGAGCCCTCAGAGA

*FLI1*: TCATCAGCCCAGCCCTCTCCACTATCAATAGAAAAGCCCGAGAGCCCTCA

||||||||||||||||||||||||| |

Patient 21 der11: TCATCAGCCCAGCCCTCTCCACTATTTTGCCATTGTTTGCTTTTGGAATC

| | |||||||||||||||||||||||||

*EWS*: GAGTTTTTGAACAGGGGGAATACTCTTTGCCATTGTTTGCTTTTGGAATC

*EWS*: AAATCCAATAGCATTTTGCAGAGTAATGTATATAATTATGGGCTCACTTC

||||||||||||||||||||||||| | | || |

Patient 22 der22: AAATCCAATAGCATTTTGCAGAGTATGGAAGGATTGTCCTGGGGCCTGAA

|||||||||||||||||||||||||||||

*FLI1*: TCAATATCAGTGCTACCCCTAAGTATGGAAGGATTGTCCTGGGGCCTGAA

*EWS*: CTTCAGAATTTAGGATCATTGCTCTTTTTAAATTACATTCTATCCACGGG

||||||||||||||||||||||||| | | |

Patient 23 der22: CTTCAGAATTTAGGATCATTGCTCTGGGAGAGGGAGAGGAAGCTTCTTCC

||| | ||| || | |||||||||||||||||||||||

*FLI1*: CTTTCTTTCTATCCATCCCTGGAGAGAGAGAGGGAGAGGAAGCTTCTTCC

*FLI1*: TCCATCCCTGGAGAGAGAGAGGGAGAGGAAGCTTCTTCCATGCCACATGG

||||||||||||||||||||||||| | ||

Patient 23 der11: TCCATCCCTGGAGAGAGAGAGGGAGCTATCCACGGGGATTTCTCAACAAC

||| || | | |||||||||||||||||||||||||

*EWS*: ATCATTGCTCTTTTTAAATTACATTCTATCCACGGGGATTTCTCAACAAC

*EWS*: CAAGGCAGGAGGATCACTTGAGACCAGGAGTTTGAGACCACCCTGGGCAA

||||||||||||||||||||||||| | | | | |

Patient 24 der22: CAAGGCAGGAGGATCACTTGAGACCTCCATGCACGCTCAGGCTTCTTCTC

| || |||||||||||||||||||||||||||

*FLI1*: TCCAGGCACCACATTCGAGAGGGCCTCCATGCACGCTCAGGCTTCTTCTC

*FLI1*: GCACCACATTCGAGAGGGCCTCCATGCACGCTCAGGCTTCTTCTCATAGC

|||||||||||||||||||||||| | | | |

Patient 24 der11: GCACCACATTCGAGAGGGCCTCCAGGAGTTTGAGACCACCCTGGGCAACA

| | |||||||||||||||||||||||||||||

*EWS*: AGGCAGGAGGATCACTTGAGACCAGGAGTTTGAGACCACCCTGGGCAACA

*EWS*: AGACATAAGAAATTAATTTCTGCATTTCCATGGACAATCTGTTGAGCTCA

|||||||||||||||||||||||||| || | ||

Patient 25 der22: AGACATAAGAAATTAATTTCTGCATTGGGCACTGGTCTCCTATTTCAGCA

| | | | |||||||||||||||||||||||||

*FLI1*: TTTAATACTCTCAATTTACCGAAGGTGGGCACTGGTCTCCTATTTCAGCA

*EWS*: TGTTTTTTTGTTGCTGTTTGTTTATATCCATTAAGGGGAAAAATGGCCAG

||||||||||||||||||||||||| || | | ||

Patient 26 der22: TGTTTTTTTGTTGCTGTTTGTTTATGGCCTGTTTACCCTGATGTGCTGGC

| || | |||||||||||||||||||||||||||

*FLI1*: GCGTCCCCACCACCACCTTCATCATGGCCTGTTTACCCTGATGTGCTGGC

*FLI1*: CGTCCCCACCACCACCTTCATCATGGCCTGTTTACCCTGATGTGCTGGCA

||||||||||||||||||||||||| | |

Patient 26 der11: CGTCCCCACCACCACCTTCATCATGATTAAGGGGAAAAATGGCCAGGCAT

| | || || |||||||||||||||||||||||||

*EWS*: TTTTTGTTGCTGTTTGTTTATATCCATTAAGGGGAAAAATGGCCAGGCAT

*EWS*: TTTGATAGCATTCTTCTTAGTATGCTTGGTAGTTTTCTTAGATTTATGAT

||||||||||||||||||||||||| | | | |||

Patient 27 der22: TTTGATAGCATTCTTCTTAGTATGCGATATCAGCATTCTGAGATTACAGA

|||| || | || |||||||||||||||||||||||||

*FLI1*: ATTGAAGTTTAATTTGAAAAGATTGGATATCAGCATTCTGAGATTACAGA

*EWS*: GAGTAAATTCAACATCGTTTTTGGCCTCCCTATCAGTCATTAATGTAAAG

||||||||||||||||||||||||| || | | | |

Patient 28 der22: GAGTAAATTCAACATCGTTTTTGGCAAACCCTGCCGGAGGCCTTCCCAGC

| | || | ||||||||||||||||||||||||||||

*FLI1*: AGAGCTTCCCTTTGTTTTTCTGGGCAAACCCTGCCGGAGGCCTTCCCAGC

*EWS*: CTGTCAAGGACAGTTTGGGAAATCCTATGTGAGCATCTACTCATAATTGC

|||||||||||||||||||||||| || | | | | ||

Patient 29 der22: CTGTCAAGGACAGTTTGGGAAATCACATATTTTGTTCAATAAAGAAACTG

| || | | ||| |||||||||||||||||||||||

*FLI1*: ACTTGAAGATGAACTATTCCAATGCTTTATTTTGTTCAATAAAGAAACTG

*FLI1*: TGAAGATGAACTATTCCAATGCTTTATTTTGTTCAATAAAGAAACTGAGG

||||||||||||||||||||||||| | | || |

Patient 29 der11: TGAAGATGAACTATTCCAATGCTTTTGTGAGCATCTACTCATAATTGCCT

| | | | || | | |||||||||||||||||||||||||

*EWS*: GTCAAGGACAGTTTGGGAAATCCTATGTGAGCATCTACTCATAATTGCCT

*EWS*: ACAAGGATTAAATGACAGTGTGACTCTAGATGATCTGGCAGACTTCTTTA

||||||||||||||||||||||||| | | | |

Patient 30 der22: ACAAGGATTAAATGACAGTGTGACTAAGGAAAGTTGATAGAGCAGAATCG

| | || | | |||||||||||||||||||||||||

*FLI1*: CCGGTGGAGGAAGTACTGCAGGGTGAAGGAAAGTTGATAGAGCAGAATCG

*EWS*: CCTCCCTATCAGTCATTAATGTAAAGTGGGGAGGCAGCTATTGCAGGCCA

||||||||||||||||||||||||| | | |

Patient 31 der22: CCTCCCTATCAGTCATTAATGTAAACTATTCCAATTTGAAAATAGATAGA

| | | | || ||||||||||||||||||||||||||||

*FLI1*: TCCCATGAGACCGCCTTTCCAGAAACTATTCCAATTTGAAAATAGATAGA

*FLI1*: TTCCAGAAACTATTCCAATTTGAAAATAGATAGAGATGGGTGGTGCCCTG

||||||||||||||||||||||||||| || | | |

Patient 31 der11: TTCCAGAAACTATTCCAATTTGAAAATAATCAGTCATTAATGTAAAGTGG

| || | || | |||||||||||||||||||||||||

*EWS*: ATTCAACATCGTTTTTGGCCTCCCTATCAGTCATTAATGTAAAGTGGGGA

*EWS*: TGCCTTGGAATTGAGGATTTTGTGATTCCAGGAGAAAGTACATCAGGCAG

||||||||||||||||||||||||| ||| | | ||

Patient 32 der22 TGCCTTGGAATTGAGGATTTTGTGAAACCATTATCCTTCCTTTCTCTAAG

|| | || | | |||||||||||||||||||||||||

*FLI1*: AGCTTGTCTGGGACACTTTCTAAGTAACCATTATCCTTCCTTTCTCTAAG

*FLI1*: GTTTTACTTCTTTGAGGATTCTTTTGAAATAGACACAAACAACAATATCT

|||||||||||||||||||| ||||| | | | | || |

Patient 32 der11: GTTTTACTTCTTTGAGGATTTTTTTGTTGTTGTTATTTAGAAGAGGTTTA

| | | || ||||||||||||||||||||||||||||||||

*EWS*: ATATGTTAGCCAGAGGGCTTTTTTTGTTGTTGTTATTTAGAAGAGGTTTA

*EWS*: ATTATCTTCAGAATTTAGGATCATTGCTCTTTTTAAATTACATTCTATCC

||||||||||||||||||||||||| || | | || | |

Patient 33 der22: ATTATCTTCAGAATTTAGGATCATTAGCAAAATTTTACAAAATACATGAC

| | | || | ||||||||||||||||||||||||||

*FLI1*: AAAACTGCAATTATAGTTGAAAAATAGCAAAATTTTACAAAATACATGAC

*FLI1*: TCTGGTTTGTGCTGACATTTCTGGCTTGTGTGACATTTCAAATCACAGAG

||||||||||||||||||||||| ||| | | || |

Patient 33 der11: TCTGGTTTGTGCTGACATTTCTGCTTTTTAAATTACATTCTATCCACGGG

|| | | | |||||||||||||||||||||||||||

*EWS*: CTTCAGAATTTAGGATCATTGCTCTTTTTAAATTACATTCTATCCACGGG

*EWS*: TGCATGCGTAGAGTTCAGCAGCCTTATAGACCAGTGTGATATTCTTGCTG

||||||||||||||||||||||||| | | ||| | | | |

Patient 34 der22: TGCATGCGTAGAGTTCAGCAGCCTTGTGGCCCATGGACAAAGCAAAACAA

|| | | || | |||||||||||||||||||||||||

*FLI1*: AGCTGCAGGCCAAATCTGGCCAAAAGTGGCCCATGGACAAAGCAAAACAA

*FLI1*: ACTAAGAAGACTGTGCAACAGAGCTTGTAGTTAGCCTATGAAGCCAAAAT

||||||||||||||||||||||| ||| | | || || | |

Patient 34 der11: ACTAAGAAGACTGTGCAACAGAGCTTGCAAGACGTGCACTAATAATATTT

| | | |||||||||||||||||||||||||||||

*EWS*: ATGTGTGTTTGTAAGGTTTGTAGCTTGCAAGACGTGCACTAATAATATTT

*EWS*: TGGTCCCATAATCCTTAAACTATAGCCTGAGGTGCACCTGATTACATAGA

||||||||||||||||||||||||| | || | |

Patient 35 der22: TGGTCCCATAATCCTTAAACTATAGGCTTTGGCAGTTTCCACTGGTGCCT

| || | ||| | ||||||||||||||||||||||||||

*FLI1*: GGAGAAATGAAAAATCAAAAAACTGGCTTTGGCAGTTTCCACTGGTGCCT

*FLI1*: TCAAGGCTTTTGGATTTAAGGTGTTGAAAATAATTTACATATTAGCTTCC

||||||||||||||||||||||||| | | | | |

Patient 35 der11: TCAAGGCTTTTGGATTTAAGGTGTTCCCAGAAGGCTTAGTTCTGTGTGTG

| | | |||| | ||||||||||||||||||||||||

*EWS*: GCTATAATATTGGTGGTTAACATAATCCAGAAGGCTTAGTTCTGTGTGTG

*EWS*: GGTCCCATAATCCTTAAACTATAGCCTGAGGTGCACCTGATTACATAGAG

||||||||||||||||||||||| | | | | |

Patient 36 der22: GGTCCCATAATCCTTAAACTATAACATTATTATTGAAAGCTAATTACAAT

| | | || |||||||||||||||||||||||||||||

*FLI1*: AGTACAAATTCTAATAGTAATTAACATTATTATTGAAAGCTAATTACAAT

*EWS:* GTTTGATAGCATTCTTCTTAGTATGCTTGGTAGTTTTCTTAGATTTATGA

||||||||||||||||||||||||| | | ||

Patient 37 der22: GTTTGATAGCATTCTTCTTAGTATGTAAGTGCTTGGATGTATCAGAGAAC

| | | | |||||||||||||||||||||||||

*FLI1*: ACTAACTCTGTGTGGGGCCCTGTTCTAAGTGCTTGGATGTATCAGAGAAC

*FLI1:*  TTCAACAAATACTTAGAGTCTACTAACTCTGTGTGGGGCCCTGTTCTAAG

||||||||||||||||| | | | | | || |

Patient 37 der11: TTCAACAAATACTTAGAACAGGGCCCCACACAGAGTATGCTTGGTAGTTT

|| | | || | |||||||||||||||||

*EWS:*  GAAGTAAACCAAAAGTTTGATAGCATTCTTCTTAGTATGCTTGGTAGTTT

*EWS*: AATAAGCCTGGTGGTAAGTTTTTGAgtattaccatagatagtgtttaaaa

||||||||||||||||||||||||| | | || | || |

Patient 38 der22: AATAAGCCTGGTGGTAAGTTTTTGATAACTCATGGAGGGTTTAAACAATA

| | || | | | |||| |||||||||||||||||||

*FLI1*: tgcacagttgttgatttcacatggtcAACTGATGGAGGGTTTAAACAATA

*EWS*: TACCTAATTTTGTTTCATCCATAGGATTTTCAGTGGAAAGAAGGGACTGA

||||||||||||||||||| | | | | ||

Patient 39 der22: TACCTAATTTTGTTTCATCAAGAAATGTAAAATCCCAAGACCACAGACTT

| | || || | | ||||||||||||||||

*FLI1*: TTGATTTGAATGGCCGTTCTAATTCAACGTGAATCCAAGACCACAGACTT

*FLI1*: TGGGATTTTACGTTTCTTGATTTGAatggccgttctaattcaacgtgaat

||||||||||| ||||||||||||| | | | ||

Patient 39 der11: TGGGATTTTACATTTCTTGATTTGACCATAGGATTTTCAGTGGAAAGAAG

||| | | ||| || |||||||||||||||||||||||||

*EWS*: ctccttttacctaattttgtttcatCCATAGGATTTTCAGTGGAAAGAAG

*EWS*: AAGCTGCTTTTGGAGACATCTTAGGAtatagaaccctataaactatatgt

|||||||||||||||||||||||||| | || || |

Patient 40 der22: AAGCTGCTTTTGGAGACATCTTAGGAAGGTCCATTctgagcacattgcat

| | | |||||||||||||||||||||||||

*FLI1*: agcatttatggaacacttatgagccAAGGTCCATTCTGAGCACATTGCAT

*FLI1*: TCTTGGATGTTATATTCATGTGACTgtctcatttctaaaatggcatccac

||||||||||||||||||||||||| || | | |

Patient 40 der11: TCTTGGATGTTATATTCATGTGACTATCAGAAAAAATGTTTTAGACTGCT

| | | | |||||||||||||||||||||||||

*EWS*: caaaaaagaaaagagatactaattgATCAGAAAAAATGTTTTAGACTGCT

*EWS*: TTTAAGAAAATAGAAAAGGAAAGGgggaaaaataatcctcctcacagaat

||||||||||||||||||||||||| | || || ||

Patient 41 der22: TTTAAGAAAATAGAAAAGGAAAGGGAAGATCTTTGAGATCTGCAAATAAA

| | || | || |||||||||||||||||||||||||

*FLI1*: tgagatgaagaccacaaatgtcatcAAGATCTTTGAGATCTGCAAATAAA

*FLI1*: GTAATTATTGAGATGAAGACCACAAAtgtcatcaagatctttgagatctg

|||||||||||||||||||||||||| | ||| || || |

Patient 41 der11: GTAATTATTGAGATGAAGACCACAAAAATAATCCTCCTCACAGAATATTT

|| | | ||| |||||||||||||||||||||||||||

*EWS*: agaaaatagaaaaggaaagggggAAAAATAATCCTCCTCACAGAATATTT

*EWS*: GAGACCCTGTCTTTTTAAAGATAAATaaagattctttaagaaaatagaaa

|||||||||||||||||||||||||| | ||| | | |

Patient 42 der22: gagaccctgtctttttaaagaTAAATCTTCTGAAAGTCAGAGTGTTAATA

|| | | | | |||||||||||||||||||||||||||

*FLI1*: ctttggctcccagataagactacAATCTTCTGAAAGTCAGAGTGTTAATA

*FLI1*: TTCTTCCCATTACTTTGGCTCCCAGAtaagactacaatcttctgaaagtc

|||||||||||||||||||||||||| || || | |

Patient 42 der11: TTCTTCCCATTACTTTGGCTCCCAGAAAAATGGCCAGGCATGGTGGCTCA

| || | | ||||||||||||||||||||||||||

*EWS*: ctgtttgtttatatccattaagggGAAAAATGGCCAGGCATGGTGGCTCA

*EWS*: CTCAAAAGAAGCTGCTTTTGGAGACATCTTAGGATATAGAACCCTATAAA

||||||||||||||||||||||||| | | |

A673 der22: CTCAAAAGAAGCTGCTTTTGGAGACCCACAGAGGAAGGACGTTTGTCTAG

| | | | || |||||||||||||||||||||||||

*FLI1*: TTACTACTTGTCTTGAATGTCAGCTCCACAGAGGAAGGACGTTTGTCTAG

*FLI1*: TTACTACTTGTCTTGAATGTCAGCTCCACAGAGGAAGGACGTTTGTCTAG

||||||||||||||||||||||||| | |

A673 der11: TTACTACTTGTCTTGAATGTCAGCTATCTTAGGATATAGAACCCTATAGA

| || | || ||||||||||||||||||||||| |

*EWS*: CTCAAAAGAAGCTGCTTTTGGAGACATCTTAGGATATAGAACCCTATAAA

*EWS*: AATTGATCAGAAAAAATGTTTTAGACTGCTAGCCCTGCTGTCTTTGGGGA

||||||||||||||||||||||||| | | || | |

RD-ES der22: AATTGATCAGAAAAAATGTTTTAGATGAGAAACTGCTGGCTCAAAGAGAT

| || | | || || ||||||||||||||||||||||||||||

*FLI1*: GAGTGGTGTTAGCCCATTTTACAGATGAGAAACTGCTGGCTCAAAGAGAT

*FLI1*: AGGGTTTACACCTGTCAATCCCAGCACTTTGGGAGGCCAAGGCGGGTGGA

||||||||||||||||||||||||||||||||| | | |

RD-ES der11: AGGGTTTACACCTGTCAATCCCAGCACTTTGGGGGAGGCCAAGGCAGGAG

| | | | ||||||||||||||||||||||||||||||||||

*EWS*: GGTGGCTCACACCTGTAATCCCAGCACTTTGGGGGAGGCCAAGGCAGGAG

*EWS*: TCCCCTTTTCCCTATCTGGTGTCTGTACTTTGATGAAGGTGAGCTATAAT

||||||||||||||||||||||||| | | ||| | | |

SKNMC der22: TCCCCTTTTCCCTATCTGGTGTCTGGGCCAATGTATAGGAAACATCATTT

| | | | | |||||||||||||||||||||||||

*FLI1*: GAGCTTAGGGCATCTTCTAGTGAAAGGCCAATGTATAGGAAACATCATTT

*EWS*: CAGTTTTGTTGAACTTAAAAGAGCCTACCTATTAAGGATGCTTTATCGTG

||||||||||||||||||||||||| || | | | | | |

TC-71 der22: CAGTTTTGTTGAACTTAAAAGAGCCCACTGCCTTATTAGAGGTGGGCCTA

| | | || ||||||||||||||||||||||||||

*FLI1*: TAAGGCAAAAGACTGAGTATCAGTCCACTGCCTTATTAGAGGTGGGCCTA

*FLI1*: GCAAAAGACTGAGTATCAGTCCACTGCCTTATTAGAGGTGGGCCTATTCC

||||||||||||||||||||||||| | | ||

TC-71 der11: GCAAAAGACTGAGTATCAGTCCACTATTAAGGATGCTTTATCGTGATGTA

||| | | |||||||||||||||||||||||||||

*EWS*: TTGTTGAACTTAAAAGAGCCTACCTATTAAGGATGCTTTATCGTGATGTA

*EWS*: ATTTCTGCATTTCCATGGACAATCTGTTGAGCTCAAGCCATCTTCTAAAA

||||||||||||||||||||||||| | || | | | | |

VH-64 der22: ATTTCTGCATTTCCATGGACAATCTCTACAGACCCTGTCTGATGGCCAGG

| | |||||||||||||||||||||||||

*FLI1*: GAAGAAATAGCAGTTCCCTGTGCCCCTACAGACCCTGTCTGATGGCCAGG

*FLI1*: TTCTTGGGGGTAATTCCCATGAGTCCTGGGAAACTCCAGGGTTTACACCT

||||||||||||||||||||| | || || || | |

VH-64 der11: TTCTTGGGGGTAATTCCCATGGCTTGAGCTCAAAGAATGGTTTTGAAACC

| | | | ||||||||||||||||||||

*EWS*: TGCCAGTATACTTCGTTGGGTCGGGGAGAACAAAGAATGGTTTTGAAACC

*EWS*: TTTGATTGATGGCACAATCTGGTTTAGAAAACTTTGTTTAGATCAATGAC

||||||||||||||||||||||||| | | |

WE-68 der22: TTTGATTGATGGCACAATCTGGTTTGGGCCTGTGGAGCACTGAGCTTCTA

| | || | | |||||||||||||||||||||||||

*FLI1*: CTCCTGGAACTCATCAACCGGCCAAGGGCCTGTGGAGCACTGAGCTTCTA

*EWS*: AATGTATATAATTATGGGCTCACTTCCTACTGGAGATGTTGAAAGTCTAA

||||||||||||||||||||||||| | | |

TC-32 der22: AATGTATATAATTATGGGCTCACTTAGTTGAAAGTTGGGAGTTTTCTCTG

|| | | | |||||||||||||||||||||||||

*FLI1*: AAAAATGAATGCTGCTTACTTTCAAAGTTGAAAGTTGGGAGTTTTCTCTG

*FLI1*: TAGAAAAAAATGAATGCTGCTTACTTTCAAAGTTGAAAGTTGGGAGTTTT

||||||||||||||||||||||||| | | || |

TC-32 der11: TAGAAAAAAATGAATGCTGCTTACTAGCCTGAGGTGCACCTGATTACATA

| | | | ||||||||||||||||||||||||||

*EWS*: TTTGGTCCCATAATCCTTAAACTATAGCCTGAGGTGCACCTGATTACATA
