# Supplementary material for: Genomic EWS-FLI1 Fusion Sequences in Ewing Sarcoma Resemble Breakpoint Characteristics of Immature Lymphoid Malignancies
Source: PLoS One. 2013 Feb 18;8(2):e56408. doi: 10.1371/journal.pone.0056408 (PMC3575406; doi:10.1371/journal.pone.0056408)
Supplement: Table S1 — Sequences of primers used in nested MLR-PCR assay. (DOC) [file pone.0056408.s004.doc]

**Table S1**

Sequences of primers used in nested MLR-PCR assay

| **der22 Primer** | **sequence 5' - 3'** | **1*** | **2*** | **position** |
| --- | --- | --- | --- | --- |
| EWS_fw 1 | AGTTACCCACCCCAAACTGGATCCTACAG | fw | ex | 19029-19057 |
| EWS_fw 2 | CAGGGGGAATACTCTTTGCCATTGTTTGC | fw | ex | 19499-19527 |
| EWS_fw 3 | CAACATCGTTTTTGGCCTCCCTATCAGTC | fw | ex | 19728-19756 |
| EWS_fw 4 | ATTTATGGAGCTCAGCCCCTAGCAGTGTG | fw | ex | 20184-20212 |
| EWS_fw 5 | GTGCCTTGGTTAGTGCCTTGGAATTGAG | fw | ex | 20469-20496 |
| EWS_fw 6 | CCAGTATACTTCGTTGGGTCGGGGAGAAC | fw | ex | 20956-20984 |
| EWS_fw 7 | CCATGGCTTTCCCCATAGGTTTTTCAAC | fw | ex | 22137-22164 |
| EWS_fw 8 | TATAAAGTTGCACCCCCAGTCCGTTGAG | fw | ex | 22906-22933 |
| EWS_fw 9 | GCTGCCCTGAAGAAAGGCATAGTGACAAG | fw | ex | 23379-23407 |
| EWS_fw 10 | TGCCGGCATTGTCTTAGGGGTTAATAAGG | fw | ex | 23702-23730 |
| EWS_fw 11 | ACCAGTGTGATATTCTTGCTGTCAAGG | fw | ex | 24234-24260 |
| EWS_fw 12 | GCCAAGCTCCAAGTCAATATAGCCAACAG | fw | in | 19057-19085 |
| EWS_fw 13 | CATCTTTAGGGCATGGCATTCCAGCTAAC | fw | in | 19545-19573 |
| EWS_fw 14 | GGCAGCTATTGCAGGCCACTATGATTTTG | fw | in | 19775-19803 |
| EWS_fw 15 | AGCCCCTAGCAGTGTGCTAAAGCCCTAAG | fw | in | 20197-20225 |
| EWS_fw 16 | TCCAGGAGAAAGTACATCAGGCAGTGGTG | fw | in | 20508-20536 |
| EWS_fw 17 | GAAACCAGAGCTTTCAGTTCCCTTCATGG | fw | in | 20998-21026 |
| EWS_fw 18 | AGTACTCAGCCGGCAGCATAATGAAAAGTG | fw | in | 22440-22469 |
| EWS_fw 19 | CTGGATGCGTCCTCTGATGTGTGTTGTAG | fw | in | 23021-23049 |
| EWS_fw 20 | TTTGGAGATGTGTTTAGCCAGTGCCCTTC | fw | in | 23425-23453 |
| EWS_fw 21 | CTCCATTTTAGCAGTGCGGGTCATTTTG | fw | in | 23966-23993 |
| EWS_fw 22 | CTTGTTGTCTCTGAAAGGCCCACCTGTAG | fw | in | 24444-24472 |
| FLI1_rv_1 | CCTGCAGTACTTCCTCCACCGGAGTGAAGG | rv | ex | 81970-81941 |
| FLI1_rv_2 | AAGAATGCTTGCCTCTCCGTCCCTGACG | rv | ex | 85424-85397 |
| FLI1_rv_3 | GCTCTCTGTTTCCTTCACACCCCATGC | rv | ex | 89356-89330 |
| FLI1_rv_4 | ACAATCTGGGTCCAGACAACCGCCCAGTG | rv | ex | 92365-92337 |
| FLI1_rv_5 | TGTGTGAGCTGACGTAGATGGGGCCTGTGG | rv | ex | 96001-95972 |
| FLI1_rv_6 | TGAATTTATGGAGGGACACGGGGCAGGG | rv | ex | 99462-99435 |
| FLI1_rv_7 | AAGAGCACTCCTTCTTTTGTGGCCTGGCAG | rv | ex | 103160-103134 |
| FLI1_rv_8 | ACCAGAGGCCAGCAACACAGCGACATCAG | rv | ex | 106499-106471 |
| FLI1_rv_9 | GGGCATTTTCTGAGATGTCTTGCAGAGGAAGTG | rv | ex | 109900-109868 |
| FLI1_rv_10 | AACAACTGTGCAGGAAGCAAAGGAGAG | rv | ex | 113371-113345 |
| FLI1_rv_11 | CAAGGAAAGACGTGCATGTTACTCCCCACTCAG | rv | ex | 115409-115377 |
| FLI1_rv_12 | AAGCACAAAGAATCAGCACCCTCTCCCCTTG | rv | in | 81856-81826 |
| FLI1_rv_13 | TCCCTCAGGCTGAAACCTCACTGCTGGAG | rv | in | 85391-85363 |
| FLI1_rv_14 | ATGGTCCTTCCCTTAGAAGAGCCTTTG | rv | in | 89315-89289 |
| FLI1_rv_15 | CGCCCAGTGTGTCTCCAAGAAAATCTG | rv | in | 92345-92319 |
| FLI1_rv_16 | ACCTAGCCCCCATACGCTGCACTCTACAAGG | rv | in | 95867-95837 |
| FLI1_rv_17 | CAGCTGGAGCATCCTGGACGTGTCTTTAAGATG | rv | in | 99429-99397 |
| FLI1_rv_18 | CCTTCTTTTGTGGCCTGGCAGGTGAGCAG | rv | in | 102766-102738 |
| FLI1_rv_19 | TGCTAAGAAGGCTGGCAGCACCTAACCCTG | rv | in | 106362-106333 |
| FLI1_rv_20 | GAGGAAGTGCAATGTTGGCAGCGGCAG | rv | in | 109876-109850 |
| FLI1_rv_21 | TGGCAAGAGGGAAAGAAATGCAGTGAG | rv | in | 113259-113233 |
| FLI1_rv_22 | CGTGCATGTTACTCCCCACTCAGGTGTCTGG | rv | in | 115399-115369 |

| **der11 Primer** | **sequence 5' - 3'** | **1*** | **2*** | **position** |
| --- | --- | --- | --- | --- |
| FLI1_fw_1 | GAAGGGAAAGAAAGAGGGACAAGGGGTGAGG | fw | ex | 78763-78793 |
| FLI1_fw_2 | GAGACTGTGGCTGGTTGGAAGTCAGTAGAG | fw | ex | 82006-82035 |
| FLI1_fw_3 | CAGATGAGAAACTGCTGGCTCAAAGAG | fw | ex | 85751-85777 |
| FLI1_fw_4 | TTTGGCTTAAAATCCGCATGGGGTGTGAAG | fw | ex | 89315-89344 |
| FLI1_fw_5 | GCTGGCCCAGCAGTGTGCAACAATCAG | fw | ex | 92797-92823 |
| FLI1_fw_6 | CTTAGCAAAAGCAGGGATGATTGCTGG | fw | ex | 96100-96126 |
| FLI1_fw_7 | TGCTTATCAGAGCTGCCCTTAAATGCACCCTG | fw | ex | 99755-99786 |
| FLI1_fw_8 | TGAACCCAGAGACTTGTCCTTTCCTGC | fw | ex | 103134-103160 |
| FLI1_fw_9 | GCTTTCGCCTTCCAACAGAGACTTTGCGG | fw | ex | 106685-106713 |
| FLI1_fw_10 | CAGAAGGGGCTGAAGGGCATGGTTGTG | fw | ex | 110310-110336 |
| FLI1_fw_11 | AACTTTGAAAGCCATGGACCTGGAGACTCCG | fw | ex | 113756-113786 |
| FLI1_fw_12 | AGAAAGAGGGACAAGGGGTGAGGGAGGCTG | fw | in | 79135-78800 |
| FLI1_fw_13 | GTCCAAGTTGGTGGCACTTCTCTGATC | fw | in | 82035-82061 |
| FLI1_fw_14 | GGAAATCAGTGGAGGGCACAAAGCTAGTCAGTG | fw | in | 85783-85815 |
| FLI1_fw_15 | TCCGCATGGGGTGTGAAGGAAACAGAGAG | fw | in | 89327-89355 |
| FLI1_fw_16 | CTGATGGATAAAAAGTCTGTCGGCTTG | fw | in | 93097-93123 |
| FLI1_fw_17 | GCGTGCTGTTTCTGTGGTCCAAAGGCAAG | fw | in | 96183-96211 |
| FLI1_fw_18 | CAAGACCCTCATAGGTGCCCATGTGCTCTG | fw | in | 99794-99823 |
| FLI1_fw_19 | CAAGACCACAGACTTGACAATCACCTG | fw | in | 103256-103282 |
| FLI1_fw_20 | TGAGGCTGAATTATCCACAATGGCTGG | fw | in | 106842-106868 |
| FLI1_fw_21 | TGGTTGTGTTTCACCCCGTCAGACTCTGTTG | fw | in | 110329-110359 |
| FLI1_fw_22 | CCTGGAGACTCCGTATTGGAGAGGAACTTGGAG | fw | in | 113774-113806 |
| EWS_rv_1 | AGGTGGGCCTTTCAGAGACAACAAGAC | rv | ex | 24468-24442 |
| EWS_rv_2 | GGCTGCTGAACTCTACGCATGCATTTACC | rv | ex | 24227-24199 |
| EWS_rv_3 | TAAGACAATGCCGGCAGTACCCATTAGTC | rv | ex | 23717-23689 |
| EWS_rv_4 | AAGGGCACTGGCTAAACACATCTCCAAAC | rv | ex | 23452-23424 |
| EWS_rv_5 | CAAATGAGGCCCAGGGTTTCCTAACAAG | rv | ex | 23117-23090 |
| EWS_rv_6 | GGGCATGTTAACAACATTGAGTGCCTTACC | rv | ex | 22297-22268 |
| EWS_rv_7 | CTCACCTCTGGAAACCATGAAGGGAACTG | rv | ex | 21040-21012 |
| EWS_rv_8 | GGAGAAAAGGTTTGCTCTTACCCCATTCC | rv | ex | 20780-20752 |
| EWS_rv_9 | GAGCCAGGTTTCTTAGGGCTTTAGCACAC | rv | ex | 20236-20208 |
| EWS_rv_10 | TCACTGCATACAACTTCCCCAAAGACAGC | rv | ex | 19720-19692 |
| EWS_rv_11 | CCATGCCCTAAAGATGTGTCCTGGATTC | rv | ex | 19560-19533 |
| EWS_rv_12 | ACCTGATCACTACTGCCCAAGGATATTCATC | rv | in | 24401-24371 |
| EWS_rv_13 | AGTGCACGTCTTGCAAGCTACAAACCTTAC | rv | in | 24075-24046 |
| EWS_rv_14 | ATGAAGGCCTTTCCCTTTAAAAGCCAGTACC | rv | in | 23514-23484 |
| EWS_rv_15 | GTCACTATGCCTTTCTTCAGGGCAGCTTG | rv | in | 23404-23376 |
| EWS_rv_16 | GCCCAGGGTTTCCTAACAAGTCACAGT | rv | in | 23109-23083 |
| EWS_rv_17 | ACCAGATAGGGAAAAGGGGATGGGAGAAG | rv | in | 21506-21478 |
| EWS_rv_18 | GACCCAACGAAGTATACTGGCAGGGAAAG | rv | in | 20975-20947 |
| EWS_rv_19 | GGTCCTGTCGGAATGAACCTGAGGAAAG | rv | in | 20596-20569 |
| EWS_rv_20 | GCTAGGGGCTGAGCTCCATAAATCAACAC | rv | in | 20206-20178 |
| EWS_rv_21 | AAAGCACTGAAATCTTCACTGGGGCATC | rv | in | 19607-19580 |
| EWS_rv_22 | CTCTTAGCAACTCACTCTGCTGCCCGTAG | rv | in | 19122-19094 |

| 1* = orientation: forward (fw), reverse (rv) |
| --- |
| 2* = external (ex), internal (in) |
