# Supplementary material for: Genomic EWS-FLI1 Fusion Sequences in Ewing Sarcoma Resemble Breakpoint Characteristics of Immature Lymphoid Malignancies
Source: PLoS One. 2013 Feb 18;8(2):e56408. doi: 10.1371/journal.pone.0056408 (PMC3575406; doi:10.1371/journal.pone.0056408)
Supplement: Table S2 — Summary of sequence motifs and repeat elements tested for association with breakpoint localization. (DOC) [file pone.0056408.s005.doc]

| **Table S2** |  |  |  |  |  |  |  |
| --- | --- | --- | --- | --- | --- | --- | --- |
| Summary of sequence motifs and repeat elements tested for association with breakpoint localization.  Number of patients with breakpoints within respective sequence elements compared to the statistical calculated numbers shown in brackets. | | | | | | | |
| * Significant difference between the two groups (t-test; p ≤ 0.05) | | | | | | | |
|  |  |  |  |  |  |  |  |
| **A** |  | | | |  |  | |
| ***EWS*** | **der22** | | | **der11** | | |  |
|
| SINE/Alu repeat | 5 |  | (4) | 3 |  | (3) |  |
| H. hypervariableminisatellite core sequence (I) | 0 |  | (0.49) | 0 |  | (0.32) |  |
| H. hypervariableminisatellite core sequence (II) | 0 |  | (0.29) | 0 |  | (0.32) |  |
| H. hypervariableminisatellite recombination seq. | 0 |  | (0.15) | 0 |  | (0.1) |  |
| chi-like sequence | 0 |  | (0.05) | 0 |  | (0.03) |  |
| Heptamer recombination signal | 0 |  | (0.04) | 0 |  | (0.03) |  |
| Nonamer recombination signal | 0 |  | (0.15) | 0 |  | (0.1) |  |
| DNA polymerase a frameshift hotspots (I) | 0 |  | (0.3) | 0 |  | (0.19) |  |
| DNA polymerase a frameshift hotspots (II) | 0 |  | (0) | 0 |  | (0) |  |
| Topoisomerase II cleavage site | 0 |  | (0.15) | 0 |  | (0.1) |  |
| human replication origin consensus sequence | 0 |  | (0.17) | 0 |  | (0.11) |  |
| Low_complexity | 0 |  | (0) | 0 |  | (0) |  |
| SINE/MIR | 0 |  | (0) | 0 |  | (0) |  |
| DNA/hAT-Charlie | 0 |  | (0) | 0 |  | (0) |  |
| LINE/L2 | 0 |  | (0) | 0 |  | (0) |  |
| translin binding sites | 0 |  | (0) | 0 |  | (0) |  |
| Palindromic sequences | 11 |  | (15.68) | 4 |  | (10.24)* |  |
| CpG islands | 0 |  | (0) | 0 |  | (0) |  |
|  |  |  |  |  |  |  |  |
|  |  |  |  |  |  |  |  |
|  |  |  |  |  |  |  |  |
| **B** |  | | | |  |  |  |
| ***FLI1*** | **der22** | | | **der11** | | |  |
|  |
| SINE/Alu repeat | 1 |  | (4) | 2 |  | (2) |  |
| H. hypervariableminisatellite core sequence (I) | 0 |  | (0) | 0 |  | (0) |  |
| H. hypervariableminisatellite core sequence (II) | 0 |  | (0.06) | 0 |  | (0.03) |  |
| H. hypervariableminisatellite recombination seq. | 0 |  | (0.02) | 0 |  | (0.01) |  |
| chi-like sequence | 0 |  | (0.17) | 0 |  | (0.09) |  |
| Heptamer recombination signal | 0 |  | (0.05) | 0 |  | (0.03) |  |
| Nonamer recombination signal | 0 |  | (0) | 0 |  | (0) |  |
| DNA polymerase a frameshift hotspots (I) | 0 |  | (0.12) | 0 |  | (0.07) |  |
| DNA polymerase a frameshift hotspots (II) | 0 |  | (0.04) | 0 |  | (0.02) |  |
| Topoisomerase II cleavage site | 0 |  | (0.05) | 0 |  | (0.03) |  |
| human replication origin consensus sequence | 0 |  | (0.03) | 0 |  | (0.02) |  |
| Low_complexity | 1 |  | (0.34) | 1 |  | (0.19) |  |
| SINE/MIR | 4 |  | (2.96) | 1 |  | (2) |  |
| DNA/hAT-Charlie | 0 |  | (0.37) | 1 |  | (0.2) |  |
| LINE/L2 | 2 |  | (2.72) | 1 |  | (1) |  |
| translin binding sites, minor | 0 |  | (0.06) | 0 |  | (0.04) |  |
| Palindromic sequences | 11 |  | (14.06) | 3 |  | (9.18)* |  |
| CpG islands | 0 |  | (0) | 0 |  | (0) |  |
